# Flexible Ti_3_C_2_T_x_-Polyurethane Electrodes for Versatile Wearable Applications

**DOI:** 10.3390/polym16182623

**Published:** 2024-09-17

**Authors:** Qiaohang Guo, Kepei Chen, Wei Yu, Man Peng, Nuozhou Yi, Zhen Wang, Peidi Zhou, Kaihuai Yang, Fei Han, Mingcen Weng

**Affiliations:** 1 School of Materials Science and Engineering, Fujian University of Technology, Fuzhou 350118, China; guoqh@fjut.edu.cn (Q.G.); nuozhouyi@smail.fjut.edu.cn (N.Y.);; 2 Institute of Smart Marine and Engineering, Fujian University of Technology, Fuzhou 350118, China; 3 School of Mechanical and Intelligent Manufacturing, Fujian Chuanzheng Communications College, Fuzhou 350007, China; 4 The Key Laboratory of Biomedical Information Engineering of Ministry of Education, School of Life Science and Technology, Xi’an Jiaotong University, Xi’an 710049, China; 5 Bioinspired Engineering and Biomechanics Center (BEBC), Xi’an Jiaotong University, Xi’an 710049, China; 6 Institute of Biology and Chemistry, Fujian University of Technology, Fuzhou 350118, China

**Keywords:** wearable electronics, Ti_3_C_2_T_x_, waterborne polyurethane, pressure sensor, proximity sensor

## Abstract

With the development of science and technology, wearable electronics are increasingly widely used in medical, environmental monitoring, and other fields. Thus, the demand for flexible electrodes is increasing. The two-dimensional material Ti_3_C_2_T_x_ has attracted much attention in the manufacture of flexible electrodes due to its excellent mechanical and electrical properties. However, the brittleness of pure Ti_3_C_2_T_x_ films has become a major obstacle for their use as flexible electrodes in wearable devices. Therefore, solving the brittleness problem of flexible electrodes based on Ti_3_C_2_T_x_ while maintaining the excellent performance of Ti_3_C_2_T_x_ has become an urgent problem. To solve this problem, Ti_3_C_2_T_x_ was compounded with waterborne polyurethane (WPU), and a Ti_3_C_2_T_x_-WPU composite film with a hierarchical structure was constructed by evaporation-assisted self-assembly. The Ti_3_C_2_T_x_-WPU composite film not only retains the excellent electrical conductivity of Ti_3_C_2_T_x_ (100 S m^−1^) but also has flexibility (20 MJ m^−3^). Furthermore, the Ti_3_C_2_T_x_-WPU composite film is applied to functional devices such as contact pressure sensors and non-contact proximity sensors. Finally, the Ti_3_C_2_T_x_-WPU composite film wearable device demonstrates its practical application potential in the field of wearable devices.

## 1. Introduction

With advancements in technology, flexible electronic devices have become a popular topic in the field of electronics [1,2,3]. Flexible electronic devices are increasingly applied across a wide range of fields, including healthcare [4], the aerospace industry, and the military, due to their superior flexibility and adaptability [5,6,7]. By optimizing the properties of materials [8], the development of deep learning systems [9] further extends the functionality and potential of flexible electronic devices, enabling them to meet ergonomic demands [10,11] while providing a new direction for the development of future smart wearable technologies. For example, OU et al. successfully prepared a dual-network-structured conductive hydrogel that can be used as a wearable strain sensor for real-time monitoring of human motion [12]. Wang et al. prepared a silk nanofiber self-powered humidity sensor that can be used for wireless monitoring systems and non-contact human-computer interaction [13]. Among these, waterborne polyurethane (WPU)-based flexible sensing materials have seen widespread application in wearable electronics, soft robotics, and biomedicine in recent years [14,15,16]. Polyurethane is an artificial polymer material with extensive applications, offering characteristics such as controllable cost, good stability, and adjustable performance compared to other polymer materials like polydimethylsiloxane, polyethylene terephthalate, and polyacrylic acid, making it an ideal substrate for flexible sensing devices. Therefore, the development of flexible sensing materials based on WPU is currently a hot topic of research. 

Conductive materials in WPU-based flexible composite sensing materials include carbon-based conductive materials, conductive metals, and conductive polymers [17,18,19]. Yao et al. proposed a skin-like strain sensor that can be used in the fields of human sound signal detection, human motion detection, and electronic skin of bionic robots using multiwalled carbon nanotubes as the conductive material [20]. After compounding with the WPU substrate, flexible sensing materials with excellent electrical conductivity and mechanical properties can be prepared. However, the aforementioned conductive fillers have some drawbacks, such as carbon-based fillers being susceptible to environmental influences, leading to poor performance and short lifespans, and metal fillers, though stable, are costly [21]. Moreover, despite extensive research on WPU-based flexible sensing materials, their sensing functions and application scenarios remain relatively singular, presenting numerous challenges in practical applications. Therefore, there is a need to develop new multifunctional WPU-based flexible sensors to expand their applications to the field of sensing and beyond. As a two-dimensional transition metal carbide, nitride, and carbonitride, Ti_3_C_2_T_x_ exhibits exceptional electrical conductivity, a high specific surface area, and excellent hydrophilicity, demonstrating significant potential for application in the realm of sensing technology [22,23,24]. The surface of Ti_3_C_2_T_x_ is enriched with functional groups such as -OH, =O, or -F, which not only facilitates its dispersion in common solvents like ethanol and water but also renders the functionalization of the Ti_3_C_2_T_x_ surface more accessible without markedly altering its conductivity [25]. The applications of Ti_3_C_2_T_x_ in the field of sensors include strain, pressure, temperature, and gas sensors, as well as the detection of various biological markers and environmental pollutants [26,27,28]. The high electrical conductivity of Ti_3_C_2_T_x_ significantly accelerates the rate of electron transfer, and the presence of surface functional groups aids in establishing stable electrode-to-target material contact in electrochemical sensors [29]. However, there are several drawbacks associated with the application of Ti_3_C_2_T_x_ in the field of sensors. Typically, the relatively poor mechanical properties and high brittleness of Ti_3_C_2_T_x_ render it challenging to withstand a certain degree of processing deformation, thereby limiting the expansion of its application domains.

Herein, WPU is selected as a toughening phase to fabricate a toughened Ti_3_C_2_T_x_-WPU composite film, thereby enhancing its mechanical processability and further expanding its application in the field of wearable devices. Ti_3_C_2_T_x_ was compounded with WPU, and a hierarchically structured Ti_3_C_2_T_x_-WPU composite film was constructed through evaporation-assisted self-assembly. The composite film retains the excellent electrical conductivity of Ti_3_C_2_T_x_ and also possesses toughness. As a well-known elastomer, WPU interacts with the soft polymer phase of Ti_3_C_2_T_x_, promoting interlayer sliding of the Ti_3_C_2_T_x_ nanosheets, thereby enhancing the toughness of the Ti_3_C_2_T_x_-WPU composite film. Furthermore, the Ti_3_C_2_T_x_-WPU composite film has been applied to functional devices such as contact pressure sensors and non-contact proximity sensors. Experimental results indicate that the contact pressure sensor based on the Ti_3_C_2_T_x_-WPU composite film exhibits a sensitivity of up to 177 kPa^−1^ and a response time of only 50 ms, demonstrating its capability to respond sensitively and rapidly to external pressure changes. In addition, the Ti_3_C_2_T_x_-WPU composite film has been applied to a non-contact proximity sensor, achieving a sensitivity of 321.4 mm^−1^, which signifies its ability to swiftly detect approaching objects. The Ti_3_C_2_T_x_-WPU composite film can be applied to soft and wearable devices, demonstrating its potential for practical applications in the domain of wearable devices.

## 2. Materials and Methods

### 2.1. Materials

Ti_3_AlC_2_ powder (200 mesh) was purchased from 11 Technology Co., Ltd. (Jilin, China). Hydrochloric acid (HCl), dimethyl sulfoxide (DMSO), lithium fluoride (LiF), polyvinyl alcohol (PVA), glycerol, and sulfuric acid (H_2_SO_4_) were purchased from Sinopharm Chemical Reagent Co., Ltd. (Shanghai, China). WPU was purchased from Shenzhen Jitian Chemical Co., Ltd. (Shenzhen, China).

### 2.2. Fabrication of the Ti_3_C_2_T_x_ Suspension

Typically, HCl, LiF, and Ti_3_AlC_2_ powders were first added into a polytetrafluoroethylene beaker under stirring and further stirred. After repeatedly washing the resultant mixture with deionized water, DMSO was added to the mixture by sonification ( sonication time was 180 min, power 660 W, room temperature). The mixture was centrifuged (10,000 rpm, 15 min) using a high-speed centrifuge to remove the supernatant and collect the sediment. Finally, deionized water was added to the centrifugal sediment with shaking to obtain the Ti_3_C_2_T_x_ suspension (5 mg mL^−1^).

### 2.3. Fabrication of Ti_3_C_2_T_x_-WPU Composites

A pipette was used to remove 2 mL of the WPU and place it in a beaker. Subsequently, 20 mL of the Ti_3_C_2_T_x_ suspension was added to the beaker. Next, the mixture was stirred with magnets on a magnetic mixer for 30 min (room temperature). When stirring was complete, the mixed solution was poured from the beaker into a petri dish. The Ti_3_C_2_T_x_-WPU mixture was placed in an oven at 60 °C and dried for 18 h. At the end of drying, the Ti_3_C_2_T_x_-WPU composites were peeled off the dish (200 μm thickness) for subsequent testing or application.

### 2.4. Fabrication of PVA-H_2_SO_4_ Hydrogel

First, 50 mL of H_2_SO_4_ (1 M) was placed in a beaker. Secondly, weigh 1 g of PVA was weighed added to the H_2_SO_4_ solution, and the above step was repeated five times. That is, a total of 5 g of PVA was added. After the fifth addition of PVA, another 50 mL of glycerol was added. After the glycerol was completely dissolved, it was poured into a petri dish and cooled to room temperature. Finally, three freeze-thaw cycles were are carried out to obtain the PVA-H_2_SO_4_ hydrogel.

### 2.5. Fabrication of Pressure Sensors Based on the Ti_3_C_2_T_x_-WPU Composite Film

First, both the PVA-H_2_SO_4_ hydrogel (5 mm thickness, thicknesses less than 5 mm are not favorable for LRC bridge recognition and greater than 5 mm the capacitance rate of change decreases, resulting in a loss of sensitivity) and the Ti_3_C_2_T_x_-WPU composite film were cut into 1 × 1 cm sizes. Second, utilizing the hydrogel’s adhesive properties, two Ti_3_C_2_T_x_-WPU composite films were pasted on the upper and lower surfaces to form a double-electrode layer structure. Finally, fine copper foils were pasted onto the upper and lower electrode surfaces.

### 2.6. Characterization and Measurements

The morphologies and microstructures of the materials were characterized using a transmission electron microscope (TEM) (JEM-2100) and a scanning electron microscope (SEM) (SU8000, JPN). The molecular compositions and phase structures were identified using a Fourier transform infrared spectrometer (FTIR) (Thermo Fisher, Nicolet 6700, USA) and an X-ray diffractometer (XRD) (Bruker Corporation, D8 advance, GER). The tensile properties of the samples were measured using a universal testing machine (Instron, 3343). The optical photos were captured by a digital camera (HUAWEI P40 Pro Plus). The electrical conductivity was measured using a Four-probe Tester (Helpass Electronic Technologies, Inc. HPS2526). The electrical signals of the pressure sensor were captured by a digital bridge (Tonghui 28320). 

## 3. Results

### 3.1. Fabrication and Characterization of the Ti_3_C_2_T_x_-WPU Composite Film

**Figure 1:**
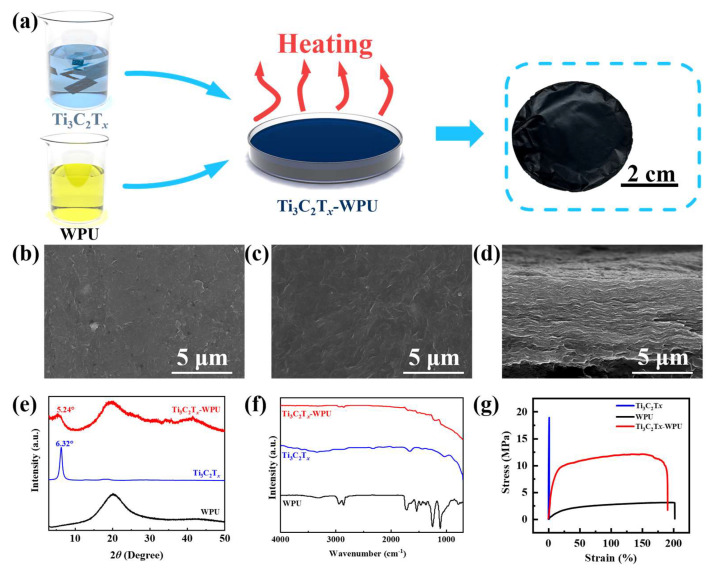
Fabrication and characterization of Ti_3_C_2_T_x_-WPU composite film. (**a**) Schematic showing the fabrication process of the Ti_3_C_2_T_x_-WPU composite film. (**b**–**d**) SEM images of the Ti_3_C_2_T_x_-WPU composite film. (**e**) XRD patterns, (**f**) FTIR spectra, and (**g**) strain-stress curves of the WPU, Ti_3_C_2_T_x_, and Ti_3_C_2_T_x_-WPU composite films.

The process of fabricating a Ti_3_C_2_T_x_-WPU composite film via an evaporation-assisted self-assembly technique is depicted in Figure 1a. Initially, the selective etching of the Al layer in the Ti_3_AlC_2_ MAX phase is conducted in a mixture of HCl/LiF, yielding Ti_3_C_2_T_x_ flakes with high electrical conductivity. The synthesis of the Ti_3_C_2_T_x_-WPU composite film utilizes an easily processable aqueous solution. After thoroughly stirring the Ti_3_C_2_T_x_ flakes and WPU, the mixture is poured into a petri dish for drying. Upon evaporation of the water, a large-sized, flexible, and conductive thin film is formed, as shown in Figure 1a. The functional groups of WPU, including the amino, carbamate, and carboxyl groups, can form multiple hydrogen bonds with the terminal groups on the surface of Ti_3_C_2_T_x_, thereby enhancing the mechanical properties of the Ti_3_C_2_T_x_-WPU composite film. The microscopic analyses using SEM are conducted to observe the morphology of the Ti_3_C_2_T_x_-WPU composite film. In Figure 1b,c, the upper and lower surface SEM images of the Ti_3_C_2_T_x_-WPU composite film reveal a defect-free and uniform film. Figure 1d shows the cross-sectional SEM image of the Ti_3_C_2_T_x_-WPU composite film; the thickness of the Ti_3_C_2_T_x_-WPU composite film is about 8 μm. In order to further verify the composition of Ti_3_C_2_T_x_-WPU composite films, detailed XRD analysis is carried out for Ti_3_C_2_T_x_, WPU, and Ti_3_C_2_T_x_-WPU composite films. As shown in Figure 1e, the analysis results show that the (002) diffraction peak of Ti_3_C_2_T_x_ is sharp and well-defined, located at 2θ = 6.32°. However, after the successful recombination of Ti_3_C_2_T_x_ and WPU, it is found that the characteristic peaks of Ti_3_C_2_T_x_ shifted significantly to the left to the position of 2θ = 5.24°. The significant shift indicates that the WPU molecule has been successfully inserted into the molecular layer of Ti_3_C_2_T_x_, resulting in a significant increase in the layer spacing [30]. The XRD patterns of WPU are then observed. It is found that it does not show the typical crystalline diffraction peak, but shows a broad peak at 2θ = 20° [31]. Interestingly, a similar peak is also observed in the XRD pattern of the Ti_3_C_2_T_x_-WPU composite film, occurring at 2θ = 20°. These results strengthen the conclusion that Ti_3_C_2_T_x_ and WPU have successfully compounded and formed a Ti_3_C_2_T_x_-WPU composite film. To investigate the interactions between Ti_3_C_2_T_x_ and WPU, FTIR spectroscopy is employed to characterize the chemical structure [30,32,33,34]. Firstly, the FTIR spectrum of the WPU film is analyzed, as shown in Figure 1f. A distinct absorption peak is observed at 2920 cm^−1^, which is attributed to the stretching vibrations of the -CH groups within the WPU molecules. A series of absorption peaks are identified in the range of 1000 to 1300 cm^−1^, corresponding to the vibrations of the C-O-C bonds in the WPU molecules. Subsequently, the FTIR spectrum of Ti_3_C_2_T_x_ has a significant absorption peak at 1630 cm^−1^ due to the -C=O groups. The stretching vibrations of the Ti-O bonds appear at 700 cm^−1^, and the -OH absorption peak is observed at 3436 cm^−1^. Finally, in the FTIR spectrum of the Ti_3_C_2_T_x_-WPU composite film, an absorption peak corresponding to the stretching vibrations of the -CH groups in WPU is identified at 2920 cm^−1^. Similarly, absorption peaks consistent with the vibrations of the C-O-C bonds in the WPU molecules are observed in the range of 1000 to 1300 cm^−1^. The characteristic stretching vibrations of the -OH, -C=O, and Ti-O bonds in Ti_3_C_2_T_x_ are also present at 3436 cm^−1^, 1630 cm^−1^, and 700 cm^−1^, respectively. Finally, mechanical tests are conducted on the Ti_3_C_2_T_x_-WPU composite film, WPU, and Ti_3_C_2_T_x_. The stress-strain curves are presented in Figure 1g. The Ti_3_C_2_T_x_-WPU composite film can endure a maximum stress of up to 12.12 MPa, a value significantly surpassing that of WPU (3.08 MPa). This indicates that the integration of Ti_3_C_2_T_x_ has enhanced the maximum stress of WPU. Furthermore, as depicted in Figure 1g, WPU exhibits a fracture strain of 200%, whereas Ti_3_C_2_T_x_ only has a fracture strain of 1.27%, indicating that Ti_3_C_2_T_x_ is prone to fracturing under external forces. The incorporation of WPU has significantly increased the maximum deformation of the Ti_3_C_2_T_x_-WPU composite film to 190%, demonstrating that the addition of WPU has substantially elevated the deformability of Ti_3_C_2_T_x_. Fracture toughness, a pivotal indicator of mechanical capability, of the Ti_3_C_2_T_x_-WPU composite film has demonstrated remarkable performance. Its fracture toughness reaches an impressive 20.73 MJ m^−3^, vastly exceeding WPU (5.01 MJ m^−3^) and the Ti_3_C_2_T_x_ (0.16 MJ m^−3^). These results unequivocally illustrate the exceptional performance of the Ti_3_C_2_T_x_-WPU composite film in resisting fractures and maintaining its structural integrity, thereby endowing it with enhanced reliability and durability for practical applications.

### 3.2. The Pressure Sensor Based on the Ti_3_C_2_T_x_-WPU Composite Film

**Figure 2:**
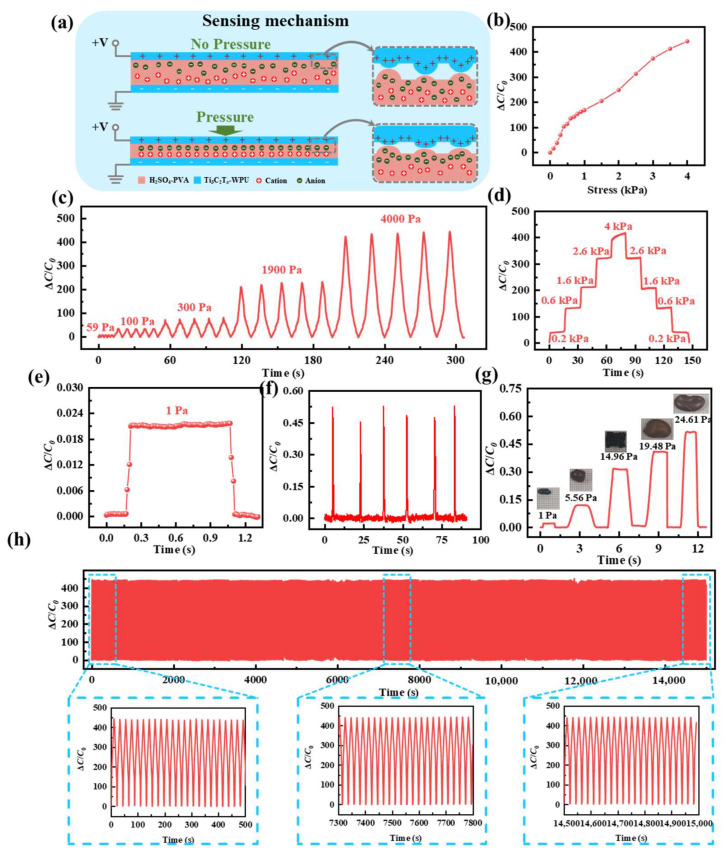
The pressure sensor based on the Ti_3_C_2_T_x_-WPU composite film. (**a**) Schematic diagram of the sensing mechanism of the Ti_3_C_2_T_x_-WPU composite film-based pressure sensor. (**b**) Sensitivity of the pressure sensor. (**c**) Dynamic step test curve of the pressure sensor. (**d**) Static step test curve of the pressure sensor. (**e**) Response and recovery curves of the pressure sensor. (**f**) The pressure sensor responds to airflow. (**g**) The pressure sensor responds to objects with different masses. (**h**) Stability of the pressure sensor.

A capacitive pressure sensor with a sandwich structure is constructed using the Ti_3_C_2_T_x_-WPU composite film as the flexible electrode and H_2_SO_4_-PVA as the ionic gel, as shown in Figure 2a. The sensing mechanism of the pressure sensor is as follows [35]. The electrons in the Ti_3_C_2_T_x_-WPU composite film and the oppositely charged ions in the H_2_SO_4_-PVA ionic gel attract each other and accumulate at their contact interface to form an electric double-layer (EDL) capacitance with high capacitance. The capacitance changes with the change in the area of the interface formed between the ions and the electrons. When no pressure is applied to its surface, the Ti_3_C_2_T_x_-WPU composite film and H_2_SO_4_-PVA ionic gel remain relatively loose. Ti_3_C_2_T_x_-WPU composite film is in contact with the H_2_SO_4_-PVA ionic gel only in a small area. Thus, the capacitance of the pressure sensor is small. The capacitance of a pressure sensor is mainly related to its electrode/electrolyte contact area. The larger the contact area, the higher the capacitance of the pressure sensor. After applying pressure to the pressure sensor, the Ti_3_C_2_T_x_-WPU composite film with a microstructure comes into contact with the H_2_SO_4_-PVA ionic gel. Thus, more EDL capacitances appear at the interface between the Ti_3_C_2_T_x_-WPU composite film and H_2_SO_4_-PVA ionic gel. The capacitance of the pressure sensor increases dramatically, resulting in high sensitivity. The sensing performance of the pressure sensor at different pressures is shown in Figure 2b. Sensitivity is defined as the ratio of the rate of capacitance change to pressure. Δ*C*/*C*_0_ of the pressure sensor increases with a gradual increase in pressure. The sensitivity of a pressure sensor is typically defined as *S*_P_ = δ(Δ*C*/*C*_0_)/δ*P*. In particular, the sensitivity is as high as 177 kPa^−1^ in the pressure range of 0–1 kPa and 97 kPa^−1^ in the pressure range of 1–4 kPa. Subsequently, the response of the pressure sensor to the dynamic pressure signal is tested and shown in Figure 2c,d. It can be seen that the pressure sensor exhibits a relatively stable and repeatable response to the same dynamic pressure. The rate of change of capacitance increases with increasing pressure. The capacitance change under different dynamic pressures is basically the same as the capacitance change under static pressures in Figure 2d, indicating that the pressure sensor also has a stable sensing ability for dynamic pressures. In addition, the response/recovery time and detection limit of the pressure sensor are also evaluated and are presented in Figure 2e. Loading 1 Pa on the pressure sensor can obtain a clear and stable response and recovery curve, demonstrating a low detection limit. However, when the pressure is 1 Pa, the response and recovery times are only 50 ms, showing a fast response/recovery time. To demonstrate the low detection limit of the pressure sensor more intuitively, two interesting demonstrations are proposed. First of all, the pressure sensor can detect the airflow generated by the ear wash ball. As presented in Figure 2f, the capacitive signal quickly returns to the initial value when the airflow disappears, which intuitively shows that the pressure sensor can respond quickly and accurately to small pressures generated by the airflow. Secondly, objects with different masses are placed on the pressure sensor in turn, and the capacitive signal of the pressure sensor is recorded and shown in Figure 2g. It is found that the pressure sensor can quickly respond to small pressures. Finally, the pressure sensor is subjected to a stability test at a pressure of 4 kPa, as shown in Figure 2h. The capacitance change rate of the pressure sensor drifts slightly in the stability test. In summary, the pressure sensor has a stable pressure sensing performance with high sensitivity, wide sensing range, fast response time, and low detection limit.

### 3.3. The Proximity Sensor Based on the Ti_3_C_2_T_x_-WPU Composite Film

**Figure 3:**
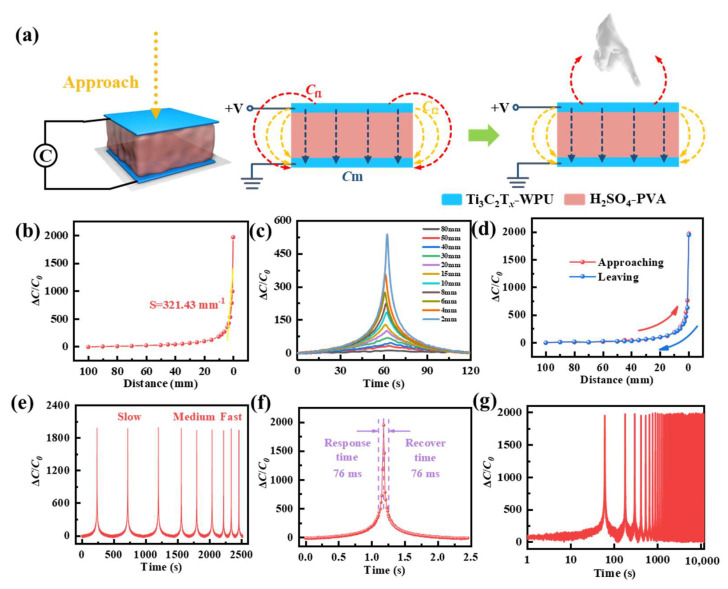
The proximity sensor based on the Ti_3_C_2_T_x_-WPU composite film. (**a**) Schematic diagram of the proximity sensing mechanism. (**b**) Distance-dependent capacitance response of the proximity sensor with different distances of aluminum (Al) block. (**c**) The time-dependent change in the capacitance of the proximity sensor at different distances between the Al block and the proximity sensor. (**d**) The capacitance response of the proximity sensor during the approaching-leaving cycle is within 100 mm. (**e**) The capacitance response of the proximity sensor during the approaching-leaving cycle at different moving speeds. (**f**) The response/recovery time of the proximity sensor. (**g**) Stability test of the proximity sensor over 800 cycles.

The mechanism of proximity sensors is mainly derived from the perturbation of the fringing electric field in the parallel-plate capacitor. During the process, two types of capacitances are defined: mutual capacitance (*C*_M_) between the upper and lower electrodes and fringing capacitance (*C*_F_) [36]. As shown in Figure 3a, when the distance between the approaching object and the proximity sensor decreases, a portion of the fringe electric field is truncated and shunted to the ground, which reduces the strength of the electric field associated with the capacitive electrodes and decreases the charge stored in the capacitor. With further proximity, charge flows out of both electrodes, resulting in an increase in C_F_ and a decrease in C_M_ [37]. Thus, the capacitance response of the proximity sensor is more significant. We specify that a target object (Al block with a base area of 1 cm^2^) approaches the proximity sensor from a distance of 100 mm from the upper surface of the proximity sensor and decelerates and stops at a distance of approximately 0.1 mm. The relationship between the Δ*C*/*C*_0_ and the proximity distance of the proximity sensor is shown in Figure 3b. The capacitance response of the proximity sensor is up to 2000 in the near range region (0–15 mm). The sensitivity of the proximity sensor is typically defined as *S*_D_ = δ(Δ*C*/*C*_0_)/δ*D*, where *D* represents the vertical distance between the proximity sensor and the object. The sensitivity is as high as 321.43 mm^−1^. As shown in Figure 3c, the proximity sensor maintains a stable capacitance response at different distances, indicating that the proximity sensor can clearly identify different response signals at different distances. In Figure 3d, we plot the process of approaching and moving away from the proximity sensor into two segments (the red curve represents the object proximity sensor and the blue curve represents the object leaving the sensor) to facilitate the analysis of the hysteresis of the proximity sensor. The capacitance response of the proximity sensor is nearly coincident when the object is approaching and leaving, indicating that the proximity sensor has good reversibility and minimal hysteresis. As shown in Figure 3e, the proximity sensor exhibits a stable capacitance response when objects are approaching at different speeds. In addition, we tested the response/recovery times of the proximity sensor, as shown in Figure 3f. The response/recovery time of the proximity sensor is only 76 ms, indicating that the proximity sensor has a fast response ability. As can be seen in Figure 3g, the stability test of the proximity sensor at the same frequency is performed for over 800 cycles at a distance of 100 mm. As can be seen in the magnified image, the capacitance amplitude fluctuates only slightly over time. This shows that the proximity sensor has good stability.

### 3.4. The Applications Based on the Ti_3_C_2_T_x_-WPU Composite Film

**Figure 4:**
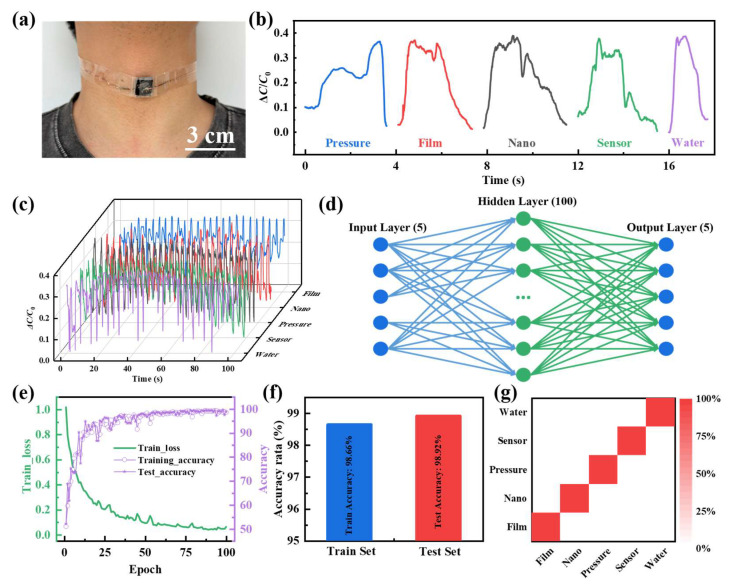
The application of pressure sensors based on the Ti_3_C_2_T_x_-WPU composite film. (**a**) The Ti_3_C_2_T_x_-WPU composite film-based pressure sensors are used in audible sound detection. (**b**) Capacitance rate of change curves for different word pronunciations. (**c**) Real-time capacitance rate of change signals from different word pronunciations. (**d**) Model structure of neural network algorithms. (**e**) Variation chart of loss and accuracy with the training cycle. (**f**) Accuracy comparison between the train and test set. (**g**) Confusion matrix for recognizing five different word pronunciations.

The monitoring of vocal cord health is critically important for maintaining human health. Unlike the bending motion of human joints, the vibration of the vocal cords is much more insignificant. Therefore, monitoring with higher accuracy and sensitivity sensors is required. Hence, the Ti_3_C_2_T_x_-WPU composite film-based pressure sensors were mounted on the skin of the larynx of a volunteer (Figure 4a), and the capacitance change curve was used to monitor the vocal folds as they emitted the “Pressure”, “Film”, “Nano”, “Sensor” and ”Water” vibrations during the pronunciation of words (Figure 4b). When the volunteer spoke the word, the sensor immediately made a capacitance response to vocal cord vibration. This shows that different words correspond to different capacitance change curves. There are also differences in the capacitance change curves for multiple pronunciations of the same word (Figure 4c), which are due to the different amplitudes of vocal fold vibration during each pronunciation. To enable accurate recognition of word pronunciation, a fully connected multilayer perceptron (MLP) neural network model with five input layers, 100 hidden layers, and five output layers is used to classify the collected word pronunciation data. The algorithmic model achieves high classification accuracy after only 100 iterations, which proves its accuracy and feasibility (Figure 4e). In the end, the accuracy of classifying and recognizing the pronunciation of the five words in the test set is as high as 98.92%, which is slightly higher than the 98.66% accuracy of the training set (Figure 4f). The confusion matrix also demonstrates the excellent classification ability of the constructed MLP neural network model for word pronunciation recognition (Figure 4g).

**Figure 5:**
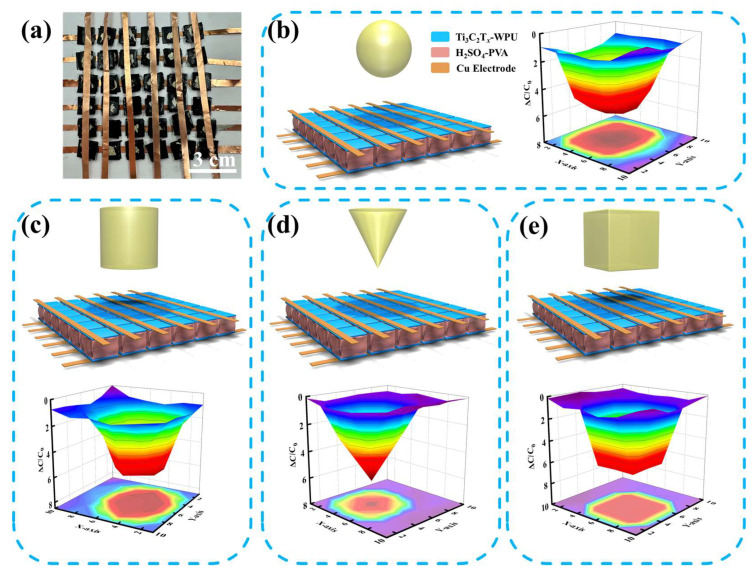
The application of proximity sensor based on the Ti_3_C_2_T_x_-WPU composite film. (**a**) The optical image of a proximity sensing array consisting of 36 sensing units. Detect various shapes such as (**b**) a sphere, (**c**) a cylinder, (**d**) a circular cone, and (**e**) a cube.

Human hands can recognize the shapes and sizes of different objects by touching and grasping them. However, the skin cannot distinguish the shape of an object without touching it. By forming a 6 × 6 array of the Ti_3_C_2_T_x_-WPU composite film-based pressure sensors (Figure 5a), the shape and size of an object can be sensed as it approaches the sensing array by detecting the capacitance response of each sensing unit, and thus exhibiting sensing capabilities that exceed the human sense of touch. When a metal ball with a diameter of 40 mm approaches the sensing array, a spherical projection corresponding to the sensing unit produces a different capacitive response, which is able to show the hemispherical in a complete 3-dimensional contour map (Figure 5b). Three different metal shapes were designed for the measurement: a cylinder, circular cone, and cube. The mapping image of the metal cylinder is a cylinder with a circular base (Figure 5c), the metal circular cone has a protruding vertex that produces a circular cone-like mapping image (Figure 5d), and the metal cube mapping image is similar to that of the metal cylinder with a square base (Figure 5e).

## 4. Conclusions

Herein, we introduce a novel Ti_3_C_2_T_x_-WPU composite film that has been meticulously engineered to exhibit enhanced mechanical processability and broadened applicability within the domain of soft sensors. The incorporation of WPU as a toughening phase within Ti_3_C_2_T_x_ is a strategic choice that imparts the composite with superior mechanical robustness. The fabrication process of the Ti_3_C_2_T_x_-WPU composite film is innovatively executed through evaporation-assisted self-assembly, culminating in a hierarchically structured material. This method ensures that the composite retains the intrinsic electrical conductivity of Ti_3_C_2_T_x_, exceeding 100 S m^−1^, while also endowing it with remarkable toughness, quantified at over 20 MJ m^−3^. The interaction between WPU and Ti_3_C_2_T_x_ is pivotal because it facilitates the interlayer sliding of Ti_3_C_2_T_x_ nanosheets, thereby augmenting the toughness of the Ti_3_C_2_T_x_-WPU composite film. The synergistic effect is a testament to the material design, which leverages the inherent properties of WPU as a well-established elastomer to enhance the mechanical integrity of the Ti_3_C_2_T_x_-WPU composite film. The practical utility of the Ti_3_C_2_T_x_-WPU composite film is underscored by its successful application in the development of functional devices, such as contact pressure sensors and non-contact proximity sensors. When integrated with an ionic gel, these pressure sensors harness the EDL effect, manifesting a comprehensive pressure sensing range from 0 to 4 kPa, coupled with high sensitivity (177 kPa^−1^), expedited response and recovery times (50 ms), and an impressively low detection threshold (1 Pa). The efficacy of the pressure sensor is exemplified by its capability to capture and discern vocal cord sounds with a high degree of precision, achieving an accuracy rate of 98.9%. Additionally, the application of the Ti_3_C_2_T_x_-WPU composite film in a non-contact proximity sensor has demonstrated a sensitivity of 321.4 mm^−1^, which underscores its proficiency in the rapid detection of the proximity and shape of approaching objects. The material design and proof-of-concept demonstrations of the Ti_3_C_2_T_x_-WPU composite film in various device components not only validate its efficacy but also illuminate the prospective opportunities for its integration into a spectrum of WPU-based soft sensors. The material design and proof-of-concept demonstrations of Ti_3_C_2_T_x_-WPU composite film in various device components highlight the future opportunity in all WPU-based soft sensors.

## Data Availability

Data are contained within the article.

## References

[1] Han F., Ge P., Wang F., Yang Y., Chen S., Kang J., Ren Y., Liu H., Wei Z., He Y. (2024). Smart Contact Lenses: From Rational Design Strategies to Wearable Health Monitoring. Chem. Eng. J..

[2] Han F., Xie X., Wang T., Cao C., Li J., Sun T., Liu H., Geng S., Wei Z., Li J. (2023). Wearable Hydrogel-Based Epidermal Sensor with Thermal Compatibility and Long Term Stability for Smart Colorimetric Multi-Signals Monitoring. Adv. Healthc. Mater..

[3] Torres Alonso E., Rodrigues D.P., Khetani M., Shin D., De Sanctis A., Joulie H., de Schrijver I., Baldycheva A., Alves H., Neves A.I.S. (2018). Graphene Electronic Fibres with Touch-Sensing and Light-Emitting Functionalities for Smart Textiles. NPJ Flex. Electron..

[4] Ray T.R., Choi J., Bandodkar A.J., Krishnan S., Gutruf P., Tian L., Ghaffari R., Rogers J.A. (2019). Bio-Integrated Wearable Systems: A Comprehensive Review. Chem. Rev..

[5] Chen J., Liu F., Abdiryim T., Liu X. (2024). An Overview of Conductive Composite Hydrogels for Flexible Electronic Devices. Adv. Compos. Hybrid Mater..

[6] Hou S., Chen C., Bai L., Yu J., Cheng Y., Huang W. (2024). Stretchable Electronics with Strain-Resistive Performance. Small.

[7] Chen S., Wu Z., Chu C., Ni Y., Neisiany R.E., You Z. (2022). Biodegradable Elastomers and Gels for Elastic Electronics. Adv. Sci..

[8] Xu J., Chang L., Chen T., Ren T., Zhang Y., Cai Z. (2023). Study of the Bending Properties of Variable Stiffness Chain Mail Fabrics. Compos. Struct..

[9] Zhou Y., Xie J., Zhang X., Wu W., Kwong S. (2024). Energy-Efficient and Interpretable Multisensor Human Activity Recognition via Deep Fused Lasso Net. IEEE Trans. Emerg. Top. Comput. Intell..

[10] Wang X., Zhang R., Miao Y., An M., Wang S., Zhang Y. (2024). PI2-Based Adaptive Impedance Control for Gait Adaption of Lower Limb Exoskeleton. IEEE/ASME Trans. Mechatron..

[11] Wang K., Boonpratatong A., Chen W., Ren L., Wei G., Qian Z., Lu X., Zhao D. (2023). The Fundamental Property of Human Leg During Walking: Linearity and Nonlinearity. IEEE Trans. Neural Syst. Rehabil. Eng..

[12] Ou K., Wang M., Meng C., Guo K., Shariar Emon N., Li J., Qi K., Dai Y., Wang B. (2024). Enhanced Mechanical Strength and Stretchable Ionic Conductive Hydrogel with Double-Network Structure for Wearable Strain Sensing and Energy Harvesting. Compos. Sci. Technol..

[13] Wang Z., Yi N., Zheng Z., Zhou J., Zhou P., Zheng C., Chen H., Shen G., Weng M. (2024). Self-Powered and Degradable Humidity Sensors Based On Silk Nanofibers and its Wearable and Human–Machine Interaction Applications. Chem. Eng. J..

[14] Wang D., Yu H., Jiang L., Qi D., Zhang X., Chen L., Lv W., Xu W., Tam K.C. (2021). Flexible, Anti-Damage, and Non-Contact Sensing Electronic Skin Implanted with MWCNT to Block Public Pathogens Contact Infection. Nano Res..

[15] Wang Y., Shu R., Zhang X. (2023). Strong, Supertough and Self-Healing Biomimetic Layered Nanocomposites Enabled by Reversible Interfacial Polymer Chain Sliding. Angew. Chem. Int. Ed..

[16] Yu C., Li X., Yang X., Qiu X., Zhang X., Chen Z., Luo Y. (2024). Dynamic Covalent Bonded Gradient Structured Actuators with Mechanical Robustness and Self-Healing Ability. Small.

[17] Burmistrov I., Gorshkov N., Ilinykh I., Muratov D., Kolesnikov E., Anshin S., Mazov I., Issi J.P., Kusnezov D. (2016). Improvement of Carbon Black Based Polymer Composite Electrical Conductivity with Additions of MWCNT. Compos. Sci. Technol..

[18] Kang H., Buchman J.T., Rodriguez R.S., Ring H.L., He J., Bantz K.C., Haynes C.L. (2019). Stabilization of Silver and Gold Nanoparticles: Preservation and Improvement of Plasmonic Functionalities. Chem. Rev..

[19] Li Y., Shi L., Cheng Y., Wang R., Sun J. (2023). Development of Conductive Materials and Conductive Networks for Flexible Force Sensors. Chem. Eng. J..

[20] Yao Z., Feng H., Shang K., Deng X., Yang T. (2023). Skin-Like Strain Sensors Based On Multiwalled Carbon Nanotube/Polydimethylsiloxane Composite Films. ACS Appl. Nano Mater..

[21] Hofmann A.I., Cloutet E., Hadziioannou G. (2018). Materials for Transparent Electrodes: From Metal Oxides to Organic Alternatives. Adv. Electron. Mater..

[22] Qin R., Nong J., Wang K., Liu Y., Zhou S., Hu M., Zhao H., Shan G. (2024). Recent Advances in Flexible Pressure Sensors Based on MXene Materials. Adv. Mater..

[23] Qin R., Shan G., Hu M., Huang W. (2021). Two-Dimensional Transition Metal Carbides And/Or Nitrides (MXenes) and their Applications in Sensors. Mater. Today Phys..

[24] Wang Y., Yue Y., Cheng F., Cheng Y., Ge B., Liu N., Gao Y. (2022). Ti_3_C_2_ T_x_ MXene-Based Flexible Piezoresistive Physical Sensors. ACS Nano.

[25] Li G., Lian S., Wang J., Xie G., Zhang N., Xie X. (2023). Surface Chemistry Engineering and the Applications of MXenes. J. Mater..

[26] Echols I.J., An H., Zhao X., Prehn E.M., Tan Z., Radovic M., Green M.J., Lutkenhaus J.L. (2020). PH-Response of polycation/Ti_3_C_2_T_x_ MXene Layer-By-Layer Assemblies for Use as Resistive Sensors. Mol. Syst. Des. Eng..

[27] Guo W., Ma Z., Chen Z., Hua H., Wang D., Elhousseini Hilal M., Fu Y., Lu P., Lu J., Zhang Y. (2024). Thin and Soft Ti_3_C_2_T_x_ MXene Sponge Structure for Highly Sensitive Pressure Sensor Assisted by Deep Learning. Chem. Eng. J..

[28] Waheed W., Anwer S., Khan M.U., Sajjad M., Alazzam A. (2024). 2D Ti_3_C_2_T_x_-MXene Nanosheets and Graphene Oxide Based Highly Sensitive Humidity Sensor for Wearable and Flexible Electronics. Chem. Eng. J..

[29] Zhang S., Zahed M.A., Sharifuzzaman M., Yoon S., Hui X., Chandra Barman S., Sharma S., Yoon H.S., Park C., Park J.Y. (2021). A Wearable Battery-Free Wireless and Skin-Interfaced Microfluidics Integrated Electrochemical Sensing Patch for On-Site Biomarkers Monitoring in Human Perspiration. Biosens. Bioelectron..

[30] Jiao C., Deng Z., Min P., Lai J., Gou Q., Gao R., Yu Z., Zhang H. (2022). Photothermal Healable, Stretchable, and Conductive MXene Composite Films for Efficient Electromagnetic Interference Shielding. Carbon.

[31] Weng M., Zhou J., Ye Y., Qiu H., Zhou P., Luo Z., Guo Q. (2023). Self-Chargeable Supercapacitor Made with MXene-bacterial Cellulose Nanofiber Composite for Wearable Devices. J. Colloid Interface Sci..

[32] Zhao J., Wang Z., Xu S., Wang H., Li Y., Fang C. (2023). Flexible Bilayer Ti_3_C_2_T_x_ Mxene/Cellulose Nanocrystals/Waterborne Polyurethane Composite Film with Excellent Mechanical Properties for Electromagnetic Interference Shielding. Colloids Surf. A Physicochem. Eng. Asp..

[33] Cheng H., Zuo T., Chen Y., Yu D., Wang W. (2023). High Sensitive, Stretchable and Weavable Fiber-Based PVA/WPU/MXene Materials Prepared by Wet Spinning for Strain Sensors. J. Mater. Sci..

[34] Wang Z., Wang L., Chang R., Shi M., Sun D. (2023). Construction of Alternating Multilayer MXene/WPU Thin Films with Excellent EMI Shielding Performance and Mechanical Properties. J. Alloys Compd..

[35] Tang Y., Wang P., Li G., Wang G., Yu W., Meng C., Guo S. (2023). Flexible and Ultra-Sensitive Planar Supercapacitive Pressure Sensor Based on Porous Ionic Foam. Adv. Eng. Mater..

[36] Niu H., Li H., Zhang Q., Kim E.S., Kim N.Y., Li Y. (2024). Intuition-and-Tactile Bimodal Sensing Based on Artificial-Intelligence-Motivated All-Fabric Bionic Electronic Skin for Intelligent Material Perception. Small.

[37] Niu H., Li H., Li N., Niu H., Li Y., Gao S., Shen G. (2024). Fringing-Effect-Based Capacitive Proximity Sensors. Adv. Funct. Mater..

